# To: Measurement of intracranial pressure and short-term outcomes of patients with traumatic brain injury: a propensity-matched analysis

**DOI:** 10.5935/0103-507X.20160036

**Published:** 2016

**Authors:** Daniel Agustín Godoy, Mario Di Napoli

**Affiliations:** 1 Intensive Care Unit, San Juan Bautista Hospital - Catamarca, Argentina and Neurointensive Care Unit, Sanatorio Pasteur - Catamarca, Argentina.; 2 Neurological Service, San Camillo de' Lellis General Hospital - Rieti, Italy and Neurological Section, SMDN-Center for Cardiovascular Medicine and Cerebrovascular Disease Prevention - Sulmona, L'Aquila, Italy.

"All science is measurement"

Ferdinand von Helmholtz (1821-1894)

To the Editor

I read with great interest the work of Ferreira et al., which deserves some reflections and commentary. First, the findings should be interpreted with great caution. Although the statistical methodology is valid and correct, it has many limitations that have been explained perfectly both by the authors and in Prof. Biestro's editorial comments.^(1,2)^ Second, severe traumatic brain injury is a heterogeneous, dynamic and evolutionary entity. Thus, it is not possible to "put in the same bag'' all of the different lesion types and clinical imaging categorization, which are clear factors that contribute to a misinterpretation of the results.^(3)^ Third, no one method of monitoring by itself can influence the outcome of a particular entity. It is the process of collecting, broadly analyzing and, finally, making choices based on data that will ultimately have a real impact.^(4)^ When the authors introduce and then discuss their paper, they engage in a major misconception that is worth mentioning. The Best Trip Trial,^(5)^ despite being published in a high-impact journal, has significant limitations. It has been subject to various criticisms and has raised controversy of all kinds.^(6-9)^ The study was not designed to assess intracranial pressure (ICP) monitoring; instead, the aim of the trial was to analyze two different approaches to the same problem: intracranial hypertension.^(4)^ However, Ferreira et al. did not include any data related to ICP measurements. In my opinion, to achieve statistical power, the study's sample size (n) should account for the number of episodes of ICP increase, not the number of patients. Fourth, the previous progress in understanding the pathophysiology of severe traumatic brain injury using modern multimodal monitoring techniques allows us today to reflect on some issues. Several questions remain unanswered. When should therapy for intracranial hypertension begin? Which measurements to treat according to lesion type? What is the period of evolution of the pathology? Which variables can influence its measurements?

It is important to note and understand that ICP is only another number in a large equation, and its control is one way, but not the only way, to achieve the final goal.^(10,11)^ Although there are other issues to analyze and reflect on, I would like to make one last point. Konstantin Tsiolkovsky, a Soviet physicist and fervent believer in the presence of life on other planets, coined the phrase ''the absence of evidence does not mean evidence of absence''.^(12)^ It has been over 60 years since the close correlation between intracranial hypertension and death was established in cases of severe traumatic brain injury.^(13)^ Since then, a significant amount of ''water has passed under the bridge'', and although we do not have high-quality studies that follow the standards of evidence-based medicine, the published data are overwhelming. Thus, until today, there have not been valid reasons to question the role of ICP monitoring in the management of severe traumatic brain injury.^(13)^

Daniel Agustín Godoy

Intensive Care Unit, San Juan Bautista Hospital - Catamarca, Argentina and Neurointensive Care Unit, Sanatorio Pasteur - Catamarca, Argentina.

Mario Di Napoli

Neurological Service, San Camillo de' Lellis General Hospital - Rieti, Italy and Neurological Section, SMDN-Center for Cardiovascular Medicine and Cerebrovascular Disease Prevention - Sulmona, L'Aquila, Italy.
